# Computed Tomography-Guided and Magnetic Resonance-Guided Adaptive Radiotherapy for Gynecological Stereotactic Body Radiotherapy Treatments: A Dosimetry and Workflow Analysis

**DOI:** 10.7759/cureus.109751

**Published:** 2026-05-27

**Authors:** Dishane C Luximon, Rojine T Ariani, John Charters, Dylan O'Connell, Puja Venkat

**Affiliations:** 1 Department of Radiation Oncology, University of California, Los Angeles, Los Angeles, USA

**Keywords:** endometrial cancer, endometrial cancer (ec), online adaptive radiotherapy (online-art), radiotherapy workflow, stereotactic body radiotherapy (sbrt), uterine cervical cancer

## Abstract

Purpose: To evaluate the dosimetric benefit of online adaptive radiotherapy (oART) for adjuvant gynecological stereotactic body radiotherapy (SBRT) and to compare plan quality and workflow efficiency between computed tomography-guided adaptive radiotherapy (CTgART) and magnetic resonance-guided adaptive radiotherapy (MRgART) platforms.

Materials and methods: Ten patients (50 fractions) treated between September 2024 and October 2025 were analyzed (CTgART: 35 fractions; MRgART: 15 fractions). Each adapted plan was compared with its corresponding non-adapted plan recalculated on the anatomy of the day. Wilcoxon signed-rank tests (W) were used to determine statistical significance (p < 0.05). Adapted plans between CTgART and MRgART were compared dosimetrically using Mann-Whitney U tests (U) for statistical significance (p < 0.05). For each comparison, the dose constraints defined by the trial protocol were used as a baseline. Timestamps from each adaptive system were extracted from the system logs and compared for each fraction.

Results: Daily adaptation substantially improved paravaginal planning target volume (PTV) coverage (V28.5Gy_mean_: 98.0% vs. 88.2%, W = -1205, p < 0.01) and clinical target volume (CTV) coverage (V30Gy_mean_: 98.2% vs. 82.0%, W = -1189, p < 0.01), with 72% (36/50 fractions) of non-adaptive fractions failing the target goals. Adaptive planning also substantially improved bowel sparing (V25Gy_mean_: 27.4cc vs. 56.1cc, W = 1207, p < 0.01). Between the CTgART and MRgART platforms, paravaginal target coverage was comparable. CTgART achieved higher nodal PTV coverage (V28.5Gy_mean_: 95.4% vs. 90.7%, U = 89.5, p < 0.01), while MRgART demonstrated improved bladder sparing (V20Gy_mean_: 23.4% vs. 43.4%, U = 15, p < 0.01). The CTgART workflow was more efficient (average completion time of 49.4 vs. 91.9 minutes) and had fewer interruptions (5.7% vs. 33.3%).

Conclusions: Online adaptation is essential to consistently meet protocol targets in gynecological SBRT, enhancing both target coverage and organs at risk sparing. While CTgART and MRgART can both achieve clinically acceptable plans, the CTgART workflow currently demonstrates superior workflow efficiency.

## Introduction

Adjuvant pelvic radiation therapy plays a critical role in improving outcomes for patients with endometrial and cervical cancers [1,2]. Standard practice targets the vaginal cuff, upper vagina, and pelvic lymph nodes, with a dose of 45-50.4 Gy delivered daily in 25-28 fractions. However, the length of the standard treatment course can be a significant burden to the patient, both in terms of time and cost [3]. As a result, there has been growing interest in establishing a hypofractionated radiotherapy regimen that would decrease the financial and quality-of-life burden on patients.

With the deformable nature of the organs at risk (OARs) around the targets, including the bowel, rectum, and bladder, high conformity is required to limit the risk of urinary and gastrointestinal radiation toxicity. Online adaptive radiotherapy (oART) has lately surfaced as a way to manage day-to-day changes in patient anatomy, enabling treatments to be adapted in real time. The use of oART has so far proven to be of clinical and dosimetric benefit for hypofractionated and stereotactic body radiotherapy (SBRT) treatments in the management of prostate cancer, pancreatic cancer, and lymph node oligometastases, providing high local control with reduced acute toxicity compared to conventional treatments [4-6]. Zeng et al. [7], for example, have shown the potential benefits of moderately hypofractionated radiotherapy (43.35 Gy to 54.4 Gy in 17 fractions) for locally advanced cervical cancer using adjuvant oART, with the patient cohort demonstrating high response rates (100% of patients with complete response) and limited side effects. Yang et al. [8] also demonstrated the feasibility of online-adapted SBRT (30-50 Gy delivered in 5-10 fractions) for pelvic-abdominal recurrent or metastatic gynecological malignancies, showing high tumor response rates (73.3%) with only one of 15 patients suffering from severe late toxicity (enteric fistula). However, to this day, there is limited data on how adaptive approaches influence the dosimetry and clinical workflow in gynecological SBRT.

This study consists of an interim analysis where we evaluate the dosimetric and workflow implications of adaptive treatments for endometrial and cervical cancers as part of the Hypofractionated External Beam Radiotherapy with Adaptive Planning (HERA) trial (NCT06538337) [9]. The HERA trial consists of an ongoing single-arm, non-randomized phase I study investigating adjuvant pelvic SBRT delivered to a total dose of 30 Gy in five fractions, with online adaptive planning performed using either computed tomography (CT) or magnetic resonance (MR)-guided platforms. Patients in this cohort are treated on either the ETHOS System (Varian Medical Systems, Palo Alto, CA) or the ViewRay 0.35T MRIdian System (ViewRay Systems, Inc., Milpitas, CA). In this work, we will analyze the dosimetric implications of adapting daily treatments, as well as compare the dosimetry and workflow of the two adaptive systems.

## Materials and methods

Summary and clinical goals of the HERA trial

The HERA trial consists of delivering 30 Gy in five fractions to the paravaginal planning target volume (PTV) and the pelvic lymph nodes PTV, with each fraction occurring every other weekday. Both the paravaginal PTV and nodal PTV were created using a 3 mm isotropic expansion of the corresponding clinical target volume (CTV). An internal target volume (ITV) technique is not utilized as a new plan is created daily, accounting for anatomical changes. For each fraction, the target volumes and OAR contours are adjusted to match the daily anatomy based on either the cone beam CT (CBCT) or 0.35T MRI. The plan is thereby adapted based on the new contours, and a mandatory plan evaluation is performed prior to delivery. The treatment planning dose constraints used in this study are shown in Table 1.

**Table 1:** Target volume and organs at risk (OAR) dose constraints for the HERA trial. HERA: Hypofractionated External Beam Radiotherapy with Adaptive Planning; PTV: planning target volume; CTV: clinical target volume.

Target/OAR	Dose constraint
Paravaginal PTV	V2850 cGy (90%) >95%
Paravaginal CTV	V3000 cGy (100%) > 95%
Nodal PTV	V2850 cGy (90%) >95 or V2613 cGy (87%) > 95% to meet bowel constraints
Paravaginal and nodal PTV	D0.035 cc < 3450 cGy (115%)
Rectum	D0.035 cc < 32 Gy
	V20 Gy < 60%, V27.5 Gy < 45%, V29 Gy < 20%
Bowel loop	D0.035 cc < 32 Gy
	V25 Gy < 40 cc
Bladder	D0.035 cc < 32 Gy
	V30 Gy < 20%, V25 Gy < 55%, V20 Gy < 60%
Femoral heads	V20 Gy < 10 cc

For this study, the data from each delivered plan at the University of California, Los Angeles (UCLA) Medical Center between September 2024 and October 2025 (number of patients = 10) were extracted using an IRB-approved protocol (23-001976; initial approval date: June 10th 2024). This included the log file of the daily adaptive treatments to extract relevant timestamps for the workflow comparison, and relevant dose-volume histograms calculated during each adaptive treatment fraction. The cohort consisted of patients with histologically confirmed endometrial (FIGO (Fédération Internationale de Gynécologie et d'Obstétrique) stage IA-IVB) or cervical cancer (FIGO stage IA-IIA) [10], meeting indications for receiving adjuvant pelvic radiotherapy alone as standard of care after surgical resection of the primary tumor. Eligibility criteria also included Karnofsky Performance Status (KPS) ≥ 60 [11] or Eastern Cooperative Oncology Group (ECOG) grade 0-2 [12], with the age of the patient ≥ 18 years old. Demographic details of the patient population are shown in Table 2.

**Table 2:** Demographic details of the patient population used in this study. IQR: interquartile range; KPS: Karnofsky Performance Status; ECOG: Eastern Cooperative Oncology Group.

Characteristic	Value (N = 10)
Age (years)	Median (IQR): 58.5 (53.5-65.75)
Sex	Female: 10 (100%)
Primary tumor site	Endometrial: 9 (90%)
	Cervical: 1 (10%)
Staging	I–II: 7 (70%)
	III–IV: 3 (30%)
Primary tumor resection performed	10 (100%)
Functional status	(N = 9) KPS median (IQR): 70 (70-80)
	(N = 1) ECOG median (IQR): 1 (1-1)

CTgART adaptive workflow

For patients treated on the CT-guided adaptive radiotherapy (CTgART) system (N = 7), the planning workflow begins with the acquisition of a planning CT (pCT) and the creation of a reference plan. All CTgART treatments in this trial are treated with a nine-equidistant-fields intensity-modulated radiotherapy (IMRT) plan using a sliding window dynamic multi-leaf collimator and are optimized by the intelligent optimization engine present in the platform [13]. At each treatment, a CBCT is acquired, and the target and OAR contours are adjusted by the physician and planner based on this daily image. The CTgART system also automatically applies the ETHOS 2.0 artificial intelligence (AI)-based auto-segmentation model [14] to the daily image to produce the OAR contours, which are then adjusted by the physician and planner. While the system also allows for automated target contour generation, our institution did not make use of this model and instead propagated the CTV contours from the pCT to the daily CBCT using a rigid registration before adjustments by the physician.

Plan optimization and dose calculation are then performed on either a synthetic CT (sCT, i.e., pCT deformed to the daily CBCT) or directly on the daily CBCT (HyperSight only). Prior to November 2025, only the sCT was used in our adaptive workflow (20 of 35 fractions). Following the installation of Hypersight, the two methods were used interchangeably, with Hypersight being the primary method. Two plans are then automatically generated during each session: a recalculated initial reference plan on the daily CBCT (or sCT), termed the “scheduled plan,” and a newly optimized plan based on the daily CBCT (or sCT), termed “adapted plan.” Those two plans are then evaluated by the physician, and the selected plan is delivered to the patient for that fraction. A second CBCT is acquired prior to beam delivery for patient setup verification.

MRgART adaptive workflow

For patients treated on the MR-guided adaptive radiotherapy (MRgART) platform (N = 3), the planning workflow begins with the acquisition of a pCT and a simulation MRI using the 0.35T MR scanner of the MRgART system. The pCT is then deformably registered to the simulation MRI to obtain an "electron density map" for dose calculation purposes. With the target and OAR contours performed on the simulation MRI, an initial reference plan is generated and manually optimized by the planner using a 23-field step-and-shoot IMRT technique.

At each treatment, a 0.35T MRI is acquired following patient setup. Target and OAR contours are propagated to the daily MRI using deformable registration and are subsequently edited by the physician and planner. Starting from the initial reference plan (i.e., scheduled plan) as a baseline, an adapted plan is generated by the planner through manual optimization until an acceptable plan is obtained according to the physician’s judgment. The selected plan is then delivered for that fraction. During beam delivery, continuous cine MRI is used for real-time target visualization, and automatic respiratory gating is applied based on a predefined 3 mm target boundary about the paravaginal CTV to manage intrafraction motion.

Adaptive versus non-adaptive dosimetry comparison

To evaluate the necessity of oART, the dosimetry achieved by the daily “adapted plans” on the CTgART and MRgART systems was evaluated against the dosimetry achieved by the “scheduled plans,” i.e., the non-adapted plan applied to the daily anatomy. In this comparison, the baseline was set to the dose constraints set by the HERA trial. The analysis was performed on a per-fraction basis, and non-parametric, Wilcoxon matched-pairs signed rank tests (W) were used to assess statistical significance of differences between the two groups, with a p-value < 0.05 being considered statistically significant.

CTgART versus MRgART workflow comparison

The adaptive workflows of the CTgART and MRgART systems were compared using timestamps that are automatically recorded by each system’s record-and-verify platform. The total adaptive treatment time was divided into four main steps: (1) patient setup, (2) contour and planning, (3) final image verification and quality assurance (QA), and (4) treatment delivery. For each adaptive fraction, the start and end times of each of these steps were taken directly from system logs. This allowed an objective comparison of the total adaptive treatment time and the time spent in each step on each fraction. However, timestamps for one of the patients treated on the MRgART platform were not available due to the system not recording the timestamps in the prior version of the software. Additionally, the number of treatment interruptions during treatment adaptation was recorded for each fraction. In case of interruptions requiring re-adaptation, the time spent on the undelivered plan was not accounted for, and the time was reset for the re-adapted plan, which was delivered.

CTgART versus MRgART dosimetry comparison

Although both the CTgART and MRgART systems use online adaptation, they rely on different imaging modalities, treatment planning systems, and plan optimization workflows, as outlined in the Methods section. These differences may lead to variations in target coverage and organ-at-risk sparing. By comparing the dosimetry of daily adapted plans between the two systems, this study aims to evaluate whether clinically meaningful dosimetric differences exist between the two systems, which may inform system selection, clinical implementation, and workflow optimization for adaptive gynecological SBRT.

As such, the dosimetry of the daily adapted plans from each system was compared on a per-fraction basis, using the dose constraints defined by the HERA trial as baseline. As the data from the two groups included different patients, statistical differences were assessed using non-parametric, unpaired Mann-Whitney U tests (U), with a p-value < 0.05 considered statistically significant.

## Results

Adaptive versus non-adaptive dosimetry comparison

**Figure 1:**
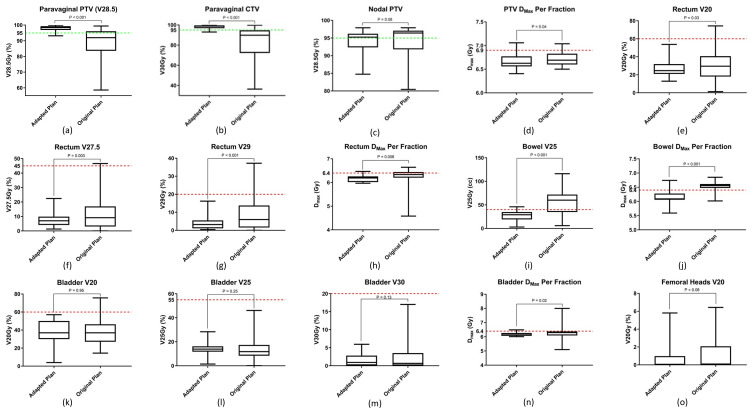
(a-o) Box-and-whisker plots for the per-fraction dosimetry comparison between adaptive treatments and non-adaptive treatments (original plan applied to the anatomy of the day) for each constraint defined in the HERA trial. Number of adapted fractions: 50; number of non-adapted fractions: 50. The box represents the 25th median and 75th percentile of the data distribution. The whiskers represent the minimum and maximum of the data distribution. The dotted line shows the dose constraint defined by the HERA trial (green: minimum dose/volume constraint; red: maximum dose/volume constraint). Statistical test used: Wilcoxon matched-pairs signed rank tests (W). P < 0.05 is considered statistically significant. HERA: Hypofractionated External Beam Radiotherapy with Adaptive Planning; PTV: planning target volume; CTV: clinical target volume.

Significant dosimetric differences were observed at the paravaginal PTV and CTV, as shown in Table 3 and Figure 1. The adaptive treatment fractions were shown to attain superior paravaginal PTV and CTV coverage with an average of 98.0 ± 1.6% (V28.5 Gy, N = 50) and 98.2 ± 1.8% (V30 Gy, N = 50) for each respective target. In contrast, the non-adaptive plans resulted in more than 72% (36 of 50 fractions) of the treatment fractions not meeting the paravaginal target dose constraints set by the HERA trial, as shown in Figures 1A, 1B. No significant differences were found at the nodal target PTV.

**Table 3:** Dosimetry comparison between the adapted treatment fractions and the non-adaptive fractions (i.e., original plan applied to the anatomy of the day). The bold text signifies the best performing regimen (statistically significant), and the text in italics indicates that the dose constraint defined by the HERA trial was not met. Number of adaptive fractions: 50; number of non-adaptive fractions: 50; W: Wilcoxon matched-pairs signed rank test. P < 0.05 is considered statistically significant. HERA: Hypofractionated External Beam Radiotherapy with Adaptive Planning; PTV: planning target volume; CTV: clinical target volume.

	Trial protocol (Average coverage/fx)	Adaptive (Average coverage/fx ± Standard deviation), N = 50	Non-adaptive (Average coverage/fx ± Standard deviation), N = 50	Statistical significance
Paravaginal PTV (V28.5 Gy)	≥ 95%	98.0 ± 1.6%	88.2 ± 10.0%	W = -1205, P < 0.01
Paravaginal CTV (V30 Gy)	≥ 95%	98.2 ± 1.8%	82.02 ± 17.4%	W = -1189, P < 0.01
Nodal PTV (V28.5 Gy)	≥ 95%	94.0 ± 3.3%	94.2 ± 4.3%	W = 342, P = 0.08
Paravaginal and Nodal PTV (D0.035 cc)	< 6.9 Gy	6.7 ± 0.2 Gy	6.7 ± 0.1 Gy	W = 391, P = 0.05
Rectum (V20 Gy)	< 60%	26.8 ± 8.9%	31.6 ± 19.4%	W= 416, P = 0.03
Rectum (V27.5 Gy)	< 45%	7.5 ± 4.2%	12.5 ± 11.7%	W = 577, P < 0.01
Rectum (V29 Gy)	< 20%	3.8 ± 3.1%	8.9 ± 9.1%	W = 715, P < 0.01
Rectum (D0.035 cc)	< 6.4 Gy	6.2 ± 0.1 Gy	6.2 ± 0.5 Gy	W = 528, P < 0.01
Bowel loop (V25 Gy)	< 40 cc	27.5 ± 10.3 cc	56.1 ± 23.5 cc	W = 1207, P < 0.01
Bowel loop (D0.035 cc)	< 6.4 Gy	6.01 ± 0.2 Gy	6.6 ± 0.2 Gy	W = 464, P < 0.01
Bladder (V20 Gy)	< 60%	37.4 ± 12.2%	38.0 ± 13.9%	W = 13, P = 0.95
Bladder (V25 Gy)	< 55%	14.3 ± 4.5%	13.4 ± 8.5%	W = -235, P = 0.25
Bladder (V30 Gy)	< 20%	1.5 ± 1.5%	2.5 ± 3.6	W = 267, P = 0.13
Bladder (D0.035 cc)	< 6.4 Gy	6.2 ± 0.1 Gy	6.3 ± 0.4 Gy	W = 464, P = 0.02
Femoral heads (V20 Gy)	< 10 cc	1.0 ± 1.7 cc	1.1 ± 1.8 cc	W = 268, P = 0.08

As for OARs, significant differences were found with regard to bowel and rectal sparing, as shown in Table 3 and Figures 1E-1I. The adaptive plans resulted in all fractions meeting the volumetric rectal dose constraints (V20 Gy, V27.5 Gy, and V29 Gy) and all but eight fractions meeting the V25 Gy bowel dose constraint, with the maximum of the V25 Gy being 45.94 cc. The non-adaptive regimen resulted in six fractions not meeting the rectum V20 Gy constraint and in at least 74% of all fractions (37 of 50 fractions) not reaching the bowel constraints set by the HERA trial, with the V25 Gy going up to 116.2 cc. No significant dosimetric differences were found for bladder and femoral head sparing.

CTgART versus MRgART system workflow comparison

**Figure 2:**
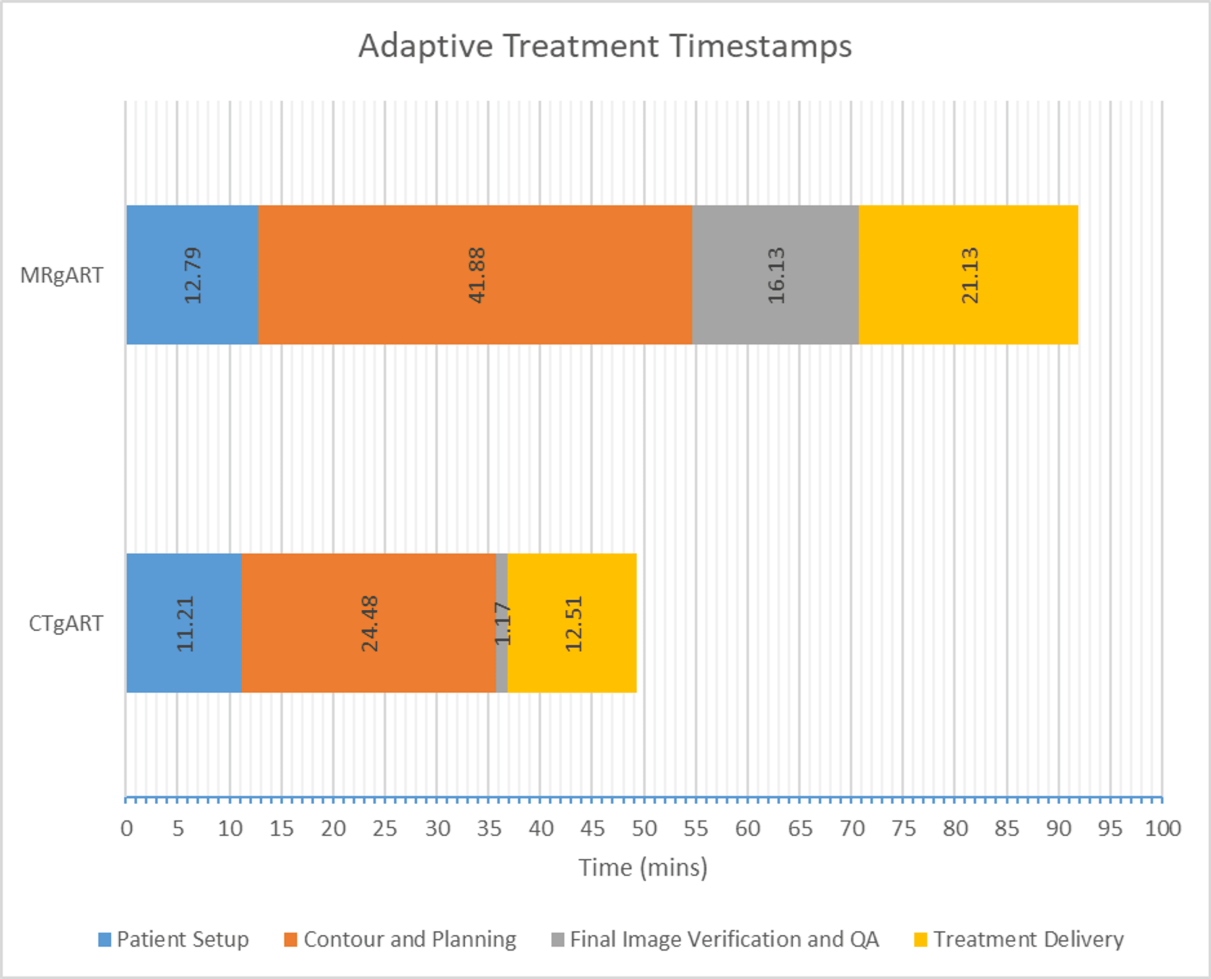
Timestamp comparison between the MRgART adaptive workflow (N = 10) and the CTgART adaptive workflow (N = 35). MRgART: magnetic resonance-guided adaptive radiotherapy; CTgART: computed tomography-guided adaptive radiotherapy.

The adaptive fractions performed on the MRgART system have an average completion time of 91.9 minutes, compared to 49.4 minutes for the CTgART system. The process showing the most discrepancy between the two systems includes the contouring and planning step (∆t = 17.4 minutes), with the final image verification and QA (∆t = 15.0 minutes) and treatment delivery (∆t = 8.62 mins) also being shorter for the CTgART system, as shown in Figure 2.

In the MRgART system, five treatment interruptions (33.3% of 15 fractions) were encountered as compared to two interruptions (5.7% of 35 fractions) in the CTgART system, for a total of seven treatment interruptions (14.0% of 50 fractions). The reasons for the treatment interruptions included patient discomfort due to bladder filling and/or pain, or changes in bladder and/or rectal filling during treatment planning and/or delivery.

CTgART versus​​​​​​​ MRgART dosimetry comparison

When comparing the adaptive plans from the MRgART platform against those from the CTgART platform, the most significant difference concerning target coverage was for the nodal PTV, with an average coverage of 95.4 ± 1.4% at V28.5 Gy (N = 35) for the CTgART against 90.73 ± 4.1% (N = 15) for the MRgART. For the MRgART, the nodal target coverage was reached on 26.7% of the fractions (four of 15 fractions) as compared to 62.8% on the CTgART (22 of 35 fractions). The dosimetry differences at the paravaginal targets were found to be statistically insignificant.

**Figure 3:**
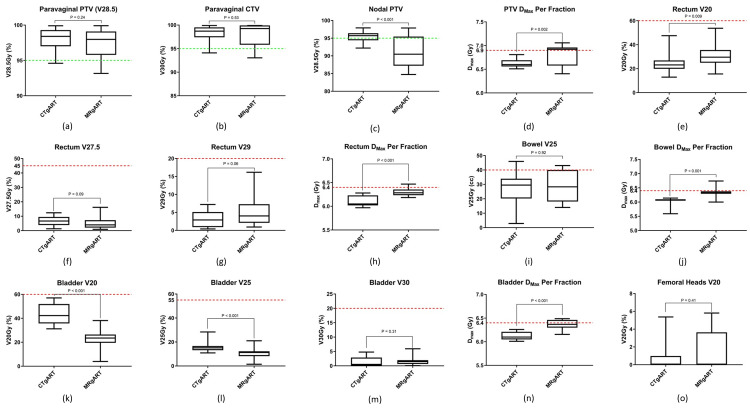
(a-o) Box-and-whisker plots for the per-fraction dosimetry comparison between the MRgART treatments and CTgART adaptive treatments for each constraint defined in the HERA trial. Number of CTgART fractions: 35; number of MRgART fractions: 15. The box represents the 25th median and 75th percentile of the data distribution. The whiskers represent the minimum and maximum of the data distribution. The dotted line shows the dose constraint defined by the HERA trial (green: minimum dose/volume constraint; red: maximum dose/volume constraint). Statistical test used: Unpaired Mann-Whitney U tests (U). P < 0.05 is considered statistically significant. HERA: Hypofractionated External Beam Radiotherapy with Adaptive Planning; PTV: planning target volume; CTV: clinical target volume; MRgART: magnetic resonance-guided adaptive radiotherapy; CTgART: computed tomography-guided adaptive radiotherapy.

Regarding OAR sparing, both systems resulted in average doses meeting the HERA trial’s constraints. However, the MRgART plans led to significantly more sparing of the bladder (20.0% improvement on average at V20 Gy) as compared to the CTgART plans. However, the CTgART plans showed better rectum sparing on average at V20 Gy (6.5% difference). Further details are shown in Table 4 and Figure 3.

**Table 4:** Dosimetry comparison between the CTgART and MRgART treatment fractions. Bold text signifies the best-performing regimen (statistically significant), and the text in italics indicates that the dose constraint defined by the HERA trial was not met. Number of CTgART fractions: 35; number of MRgART fractions: 15; U: unpaired Mann-Whitney U test. P < 0.05 is considered statistically significant. HERA: Hypofractionated External Beam Radiotherapy with Adaptive Planning; PTV: planning target volume; CTV: clinical target volume; MRgART: magnetic resonance-guided adaptive radiotherapy; CTgART: computed tomography-guided adaptive radiotherapy.

	Target constraint (Average coverage/fx)	CTgART adaptive (Average coverage/fx ± Standard deviation), N = 35	MRgART adaptive (Average coverage/fx ± Standard deviation), N = 15	Statistical significance
Paravaginal PTV (V28.5 Gy)	≥ 95%	98.2 ± 1.4%	97.5 ± 1.9%	U = 206.5, P = 0.24
Paravaginal CTV (V30 Gy)	≥ 95%	98.3 ± 1.5%	98.0 ± 2.3%	U = 232.5, P = 0.53
Nodal PTV (V28.5 Gy)	≥ 95%	95.4 ± 1.4%	90.73 ± 4.1%	U = 89.5, P < 0.01
Paravaginal and Nodal PTV (D0.035 cc)	< 6.9 Gy	6.6 ± 0.1 Gy	6.8 ± 0.2 Gy	U = 122.5, P < 0.01
Rectum (V20 Gy)	< 60%	24.8 ± 7.8%	31.3 ± 9.7%	U = 141, P < 0.01
Rectum (V27.5 Gy)	< 45%	6.7 ± 3.1%	9.3 ± 5.3%	U = 181, P = 0.09
Rectum (V29 Gy)	< 20%	3.2 ± 2.2%	5.3 ± 4.4%	U = 174, P = 0.06
Rectum (D0.035 cc)	< 6.4 Gy	6.1 ± 0.1 Gy	6.3 ± 0.1 Gy	U = 56, P < 0.01
Bowel loop (V25 Gy)	< 40 cc	27.3 ± 10.2 cc	28.0 ± 10.8 cc	U = 257, P = 0.92
Bowel loop (D0.035 cc)	< 6.4 Gy	6.0 ± 0.1 Gy	6.4 ± 0.2 Gy	U = 62, P < 0.01
Bladder (V20 Gy)	< 60%	43.4 ± 8.2%	23.4 ± 7.7%	U = 15, P < 0.01
Bladder (V25 Gy)	< 55%	15.7 ± 3.7%	11.1 ± 4.6%	U = 82, P < 0.01
Bladder (V30 Gy)	< 20%	1.4 ± 1.6%	1.8 ± 1.6%	U = 214, P = 0.31
Bladder (D0.035 cc)	< 6.4 Gy	6.1 ± 0.1 Gy	6.4 ± 0.2 Gy	U = 44, P < 0.01
Femoral heads (V20 Gy)	< 10 cc	0.9 ± 1.5 cc	1.2 ± 2.0 cc	U = 223.5, P = 0.41

It is important to note that the gating capability of the MRgART system may lead to superior target coverage and OAR sparing as compared to non-gated treatments, as suggested by prior studies [15,16]. The dosimetric impact of gating treatments was not examined in this work and falls out of the scope of this study.

## Discussion

In this study, we evaluated the dosimetric and workflow implications of adaptive treatments for gynecological adjuvant SBRT treatments performed under the HERA trial. The plan quality of the adaptive treatments was compared to the non-adaptive plan on the daily images to explore the dosimetric benefits of online adaptation. Furthermore, the workflow and plan quality of the fractions performed on a CTgART system were compared to those performed on an MRgART system.

We demonstrated that online adaptation provides substantial plan quality benefits as compared to non-adaptive plans, both in terms of target coverage and OAR sparing. While the non-adaptive plans resulted in 72% of fractions (36 of 50 fractions) not meeting the paravaginal PTV and CTV target coverage goals, the adapted plans met the target goals on 96.0% of the fractions (48 of 50 fractions). Additionally, a significant improvement in bowel and rectum sparing was observed when the plans were adapted. The dosimetric impact on oncologic and toxicity outcomes will be evaluated after more data are collected.

In terms of the dosimetry comparison between CTgART and MRgART, our results demonstrate that although both platforms produced clinically acceptable plans overall, statistically significant differences in nodal PTV coverage and OAR sparing were observed. These differences may reflect variations in the plan optimization workflow, including prioritization of OAR sparing during adaptation, and should therefore be considered when selecting an adaptive platform for gynecologic SBRT. While slight improvements in nodal PTV coverage and rectum sparing were seen on the CTgART plans, these are counteracted by the gating capability of the MRgART system and superior bladder sparing. Additionally, the increased soft tissue contrast provided by the MRgART platform makes it a good candidate to better visualize and contour the anatomy at the treatment regions for gynecological cancers.

The CTgART workflow was found to be 54% shorter compared to MRgART, which is concordant with similar comparisons performed by Price et al. for prostate, bladder, and pancreas adaptive treatments [17]. This difference is largely attributed to the AI-driven auto-contouring and auto-planning present within the CTgART platform. On the MRgART platform, while the OAR and target contours are propagated through deformable registration, they are often fully re-contoured by the physician and planner, increasing treatment planning time. Additionally, the treatment plans are manually optimized on the MRgART platform, further lengthening this process. Our results have also shown more frequent treatment interruptions on the MRgART (33.3% of 15 fractions compared to 5.7% of 35 fractions on the CTgART), which can be attributed to the longer time taken from initial scan to treatment delivery on the MRgART system. For this patient cohort, the reduced time from patient setup to treatment on the CTgART currently makes it the preferred option at our institution, as it avoids time for bladder and rectal filling and reduces patient discomfort.

One limitation of this study is that inter-observer differences of the target and OAR contours on each modality were not analyzed. Given that the contours are evaluated by an attending physician at each fraction, it was assumed that the inter-observer differences in contours minimally affected the plan quality. In future studies, we will also investigate any correlation between plan quality and radiation toxicity, as well as oncologic outcomes. This is currently out of the scope of this study due to the limited number of patients in the dataset.

Nevertheless, the results from this study may inform the selection of an adaptive radiotherapy platform and guide the design of efficient adaptive workflows. This work particularly helps clarify how differences in imaging, plan adaptation, and delivery affect both plan quality and clinical efficiency. These findings can support centers in choosing the most appropriate platform for gynecological SBRT based on their clinical goals, staffing, and patient throughput needs. In addition, understanding which steps contribute most to total adaptive time can guide future workflow innovations, which can ultimately lead to safer and more efficient adaptive radiotherapy treatments.

## Conclusions

In this work, we analyzed the dosimetric implications of adapting daily treatments, as well as compared the dosimetry and workflow of two adaptive systems used in a trial involving gynecological adjuvant SBRT treatments. We demonstrated that adapting the plans resulted in significant dosimetric gains in terms of target coverage and OAR sparing. Additionally, our results show that the CTgART and MRgART platforms can both produce clinically acceptable adaptive plans, with differences observed at the nodal target coverage, rectum sparing, and bladder sparing. Our workflow analysis shows that the CTgART platform is currently more efficient during the online adaptive workflow as compared to the current MRgART system.
